# *Chlamydia trachomatis* and Human Papillomavirus Infection in Women From Southern Hunan Province in China: A Large Observational Study

**DOI:** 10.3389/fmicb.2020.00827

**Published:** 2020-05-05

**Authors:** Hongliang Chen, Lipei Luo, Yating Wen, Bei He, Hua Ling, Jinwei Shui, Ping He, Xiaoli Hou, Shixing Tang, Zhongyu Li

**Affiliations:** ^1^Institute of Pathogenic Biology, Hengyang Medical College, Hunan Provincial Key Laboratory for Special Pathogens Prevention and Control, Hunan Province Cooperative Innovation Center for Molecular Target New Drug Study, University of South China, Hengyang, China; ^2^Department of Clinical Microbiology Laboratory, Chenzhou No. 1 People’s Hospital, Chenzhou, China; ^3^Dermatology Hospital, Southern Medical University, Guangzhou, China; ^4^Affiliated Chenzhou Hospital, Southern Medical University, Guangzhou, China; ^5^Department of Epidemiology, School of Public Health, Southern Medical University, Guangzhou, China

**Keywords:** *Chlamydia trachomatis*, human papillomavirus, coinfection, prevalence, genotype distribution

## Abstract

*Chlamydia trachomatis* and human papillomavirus (HPV) are the most common pathogens of sexually transmitted infections (STIs), which can increase the risk of cervical cancer and infertility. The purpose of this study was to evaluate the prevalence, genotype and risk factors of *C. trachomatis* and/or HPV infection in women attending the annual physical examination, assistant reproductive treatment and visiting the gynecology clinics from Southern Hunan province in China. Cervical-swab samples were collected from 5006 participants. We found that the overall prevalence of *C. trachomatis*, HPV infection and *C. trachomatis*/HPV coinfection was 4.7% (236/5006), 15.5% (778/5006) and 1.2% (59/5006), while the prevalence of asymptomatic infection of that was 3.8% (38/1006), 10.8% (109/1006) and 0.6% (6/1006), respectively. Furthermore, 25.0% (59/236) of *C. trachomatis* infection and 7.6% (59/778) of HPV infection were attributable to *C. trachomatis* and HPV coinfection. *C. trachomatis* and HPV infection were more often observed in young women of less than 25 years (10.4% and 21.3%, respectively) and in the outpatients from gynecology clinics (5.2% and 18.0%, respectively). Of note, a higher prevalence of *C. trachomatis* infection was observed in HPV-positive women (7.6%) than HPV- negative ones (4.2%), and vice versa. The top three *C. trachomatis* genotypes were E (1.4%), F (1.1%) and J (0.8%), and the counterparts of HPV genotypes were HPV52 (4.2%), HPV16 (2.3%) and HPV58 (2.2%), respectively. Among the 151 outpatients with colposcopy data, HPV infection was associated with severe cervical lesions with OR of 15.86 (95% CI 3.14–80.0, *P* < 0.001) while *C. trachomatis* infection was more likely associated with a low grade colposcopy impression (OR = 3.25, 95% CI: 1.22–8.65, *P* = 0.018). Our data highlight the high prevalence of asymptomatic *C. trachomatis* and HPV infection, particularly among women of <25 years. The two pathogens may serve as mutual risk factors to increase the risk of infections and cervical lesions. Widespread implementation of HPV and *C. trachomatis* screening programs, especially for young women, would be an effective strategy to relieve the burden of sexually transmitted infections.

## Introduction

*Chlamydia trachomatis* (CT), an obligate intracellular parasitic bacterium, is the leading bacterial cause of sexually transmitted infections (STIs) (Witkin et al., 2017) while human papillomavirus (HPV) has been documented to cause cervical cancer (Harden and Munger, 2017). It is estimated that 101 million new cases of *C. trachomatis* occur annually worldwide, causing important health problems. Actually, the number of *C. trachomatis*-infected population may be more than the reported cases since 80% of *C. trachomatis* infection may be asymptomatic and show subclinical course. This microorganism can be subdivided into at least 19 serotypes (Rawre et al., 2017; Hadfield et al., 2018). Serovars A to C are mainly related to trachoma; serovars D–K usually cause urogenital infections (Geisler et al., 2003), such as urethritis, cervicitis, pelvic inflammatory disease, and ectopic pregnancy; and serovars L1–L3 are associated with lymphogranuloma venereum.

Epidemiological studies have shown that *C. trachomatis* infection has also been identified as an independent risk factor of cervical cancer, indicating a potential relationship with HPV infection (Bellaminutti et al., 2014; Di Pietro et al., 2018). In terms of pathogenesis, *C. trachomatis* is capable of triggering chronic or recurrent infections and long-term inflammations of the urethra (Chen et al., 2019), inducing local secretion of immune mediators, enhancing the production of reactive oxygen species (ROS) and generation of free radicals, which may cause damages to host mucosal barriers and cell-mediated immunity (Conde-Ferraez et al., 2017). These biological effects of *C. trachomatis* infection could facilitate the transmission and co-infection of HPV and decrease the host’s ability to resolve HPV infection.

HPV is the most common viral infection of the reproductive tract and is also a well-established risk factor for cervical cancer (Schiffman et al., 2016; Clarke et al., 2018). To date, over 100 HPV genotypes have been identified, and are associated with cervical lesions. Infections with low-risk HPV (LrHPV) genotype, such as HPV types 6 and 11, can cause benign warts and recurrent respiratory papillomatosis. High-risk HPV (HrHPV) types are closely associated with cervical, anal, and other genital cancers (Harden and Munger, 2017). The overall HrHPV infection rate in China is over 89% in patients with cervical cancer, ranging from 81% in North to 94% in Southwest of China (Li et al., 2019).

Both HPV and *C. trachomatis* are sexually transmitted, sharing common transmission routes and the same risk factors. Several studies have found that *C. trachomatis* promotes HPV persistence, and is associated with cervical cancer (Bhatla et al., 2013). However, the effects and clinical consequences of *C. trachomatis* and HPV coinfection are poorly explored. Understanding the prevalence, genotype distribution and risk factors of *C. trachomatis* and HPV infection, especially for asymptomatic infection, are critical for effective prevention and interventions.

Since *C. trachomatis* has not yet been included as a reportable STI in the national STI surveillance program in China, the detailed epidemic data is lacking. The first nationwide population-based *C. trachomatis* infection investigation in 1999–2000 found a prevalence of 2.1% among men and 2.6% among women (Parish et al., 2003), whereas women with secondary infertility have a relatively high prevalence of 13.3% (Shi et al., 2001). Furthermore, the HPV prevalence varies significantly and ranges from 16.8–36% in China (Li et al., 2006; Ji et al., 2019). Considering the geographic variation of *C. trachomatis* and HPV infection, we thus enrolled a large number of women from Southern Hunan province in China, including healthy women attending the annual physical examination, or young women seeking reproductive assistance and outpatients in the gynecology clinics to investigate prevalence and risk factors of *C. trachomatis* and HPV infection. Such data may provide insights into the epidemiology of *C. trachomatis* and HPV infection in the region.

## Materials and Methods

### Participants and Clinical Samples

During December 2018 and November 2019, cervical-swab samples were collected for detecting *C. trachomatis* and HPV from three groups of Chinese women, i.e., the seemingly healthy women to receive annual physical examination in the physical examination center (PEC); women seeking for assistance at the assisted reproductive technology center (ART); and outpatients from the gynecology clinics (GC), respectively. The inclusion criteria are female, not taking antibiotics, not being pregnant, and no parturition when sampling. Asymptomatic *C. trachomatis*/HPV infection was defined as positive for *C. trachomatis*/HPV nucleic acid detection but without symptoms, such as painful sexual intercourse, abnormal vaginal discharge, urethritis, irregular vaginal bleeding, or bleeding after sexual intercourse and genital warts (Wiesenfeld et al., 2005; Erickson et al., 2013). Asymptomatic participants were mainly from ART. Cervical-swab samples were collected using a 200 mm polyethylene Cervix brush device (Hybribio Corp, Guangdong) following the regular procedures for speculum examination. The specimens were transferred to a tube containing cervical cell preservation solution provided in the kit and stored at room temperature for immediate processing or −80°C until analysis. Cervical samples can be kept in the transport medium for 2 weeks at 4°C according to the manufacturer’s manual.

The study was conducted in the teaching hospital, Chenzhou No.1 People’s Hospital, in Chenzhou, and was under the Principles of the Declaration of Helsinki. It was approved by The Ethics Committee of Chenzhou No.1 People’s Hospital (CZ/1128). Written inform consents were obtained from the participants. All experiments were carried out in the lab certified by the National Center for Clinical Laboratories following the laboratory biosafety guidelines.

### DNA Extraction

DNA was extracted from cervical-swabs samples within 48 h after collection using the QIAamp mini kit (Qiagen). DNA isolation and purification were conducted according to the manufacturer’s instructions. Briefly, the samples were centrifuged at 14 000 rpm for 1 min. The supernatant was removed and 20 μL protein K was added, following by incubation for 10 min at 37°C. DNA was immediately amplified by polymerase chain reaction (PCR) or stored at −20°C for further analysis. The concentration and purity of the extracted DNA were determined by using Thermo Scientific Varioskan Flash Spectral Scanning Multimode Reader (Thermo Fisher Scientific, Inc.). Water control was processed in parallel to monitor contamination during DNA extraction. The human housekeeping gene β-globin was used as an endogenous internal control to ensure the quality of DNA and the efficiency of PCR.

### HPV Genotyping

HPV detection and genotyping were performed by using the Hybribio Rapid Geno-Array test kit (Hybribio Corp, Guangdong) based on the PCR-reverse dot blot hybridization method. The low limit of detection (LOD) for HPV DNA is 500 copies/ml according to manufacturer’s protocol. The kit detects 21 HPV genotypes, including 15 HrHPV genotypes (types 16, 18, 31, 33, 35, 39, 45, 51, 52,53, 56, 58, 59, and 68), and 6 LrHPV genotypes (types 6, 11, 42, 43, 44, and CP8304 [81]). This assay uses HPV L1 consensus PCR primers (MY09/11) for the amplification. HPV genotypes were then determined through hybridization using genotype-specific probes (Ji et al., 2019).

### *C. trachomatis* Detection and Genotyping

A 200 bp conserved cryptic plasmid fragment of *C. trachomatis* DNA was amplified for diagnosis of *C. trachomatis* infection with the following primes: CT1: 5′-TTCCCCTTGTAATTCGTTGC-3′ and CT2: 5′-TAGTAACTGCCACTTCATCA-3′. PCR was carried out in 25 μl reaction mixture in a thermal cycler, the reaction conditions for which were 95°C for 5 m, followed by 35 cycles of 95°C for 50 s, 55°C for 45 s and 72°C for 45 s, with a final elongation at 72°C for 10 min as previously described (Griffais and Thibon, 1989). The cryptic plasmid-based PCR showed a LOD of 400 copies/ml and a concordance rate of 95% with a commercial *C. trachomatis* molecular diagnostic kit (Sansure Biotech, China).

For *C. trachomatis* genotyping, an approximate 1100 bp fragment encompassing VS1–VS4 of *C. trachomatis* omp1 gene was amplified using nested PCR with the following primers: outer primes *omp1* P1: 5′-CTCAACTGTAACTGCGTATTT-3′ and omp1 P2: 5′-ATGAAAAAACTCTTGAAATCG-3′. PCR was carried out in 25 μl reaction mixture with the following reaction conditions: 95°C for 5 m, followed by 25 cycles of 95°C for 60 s, 55°C for 60 s and 72°C for 80 s, with a final elongation at 72°C for 10 min. An approximate 580 bp VS1–VS2 fragment was further amplified using the second-round PCR with the inner primer sets P3: 5′- TGAACCAAGCCTTATGATCGACGGA-3′ and omp1 P2: 5′-TCTTCGAYTTTAGGTTTAGATTGA-3′, and the reaction conditions: 95°C for 5 m, followed by 35 cycles of 95°C for 30 s, 55°C for 30 s and 72°C for 30 s, with a final elongation at 72°C for 10 min (Hsu et al., 2006). The *C. trachomatis* (ATCC VR-348B) was used as a positive control and DNase-free water as a negative control in PCR. Positive PCR products for *C. trachomatis omp1* VS1–VS2 were sequenced bi-directionally in Ruibo, Beijing. Genotypes of *C. trachomatis* were determined using BLAST program^1^ as previously described (Hsu et al., 2006; Yang et al., 2010).

### Colposcopy Test

Colposcopy screening was performed using a digital electronic colposcope (SLC-3000, Philips, Shenzhen) following a standard procedure. According to the standard and terminology of the American Society for Colposcopy and Cervical Pathology (ASCCP) (Khan et al., 2017), colposcopy impression includes benign, low grade features, high grade features, and cancer. Low-grade and high-grade features correspond to Grade 1 (minor) and Grade 2 (major) abnormal colposcopy findings defined by the International Federation for Cervical Pathology and Colposcopy (IFCPC) nomenclature.

### Statistical Analysis

Statistical analyses were conducted using SPSS version 19.0 software. Chi-Square Test was used to compare frequencies of discrete variables. The Fisher Exact Test was applied when necessary. Bivariate logistic regression was used to assess the risk factors affecting the prevalence of *C. trachomatis* and HPV. Statistical differences were considered being significant when *P* < 0.05.

## Results

### Prevalence of *C. trachomatis* and HPV Infection

From December 2018 to November 2019, a total of 5027 women were enrolled in our study, and 21 participants were excluded due to poor sample quality or lack of personal information. Therefore, 5006 women with cervical swab samples were analyzed. Among them, 1006 women were from PEC, 666 from ART and 3334 outpatients from GC (Table 1). The overall prevalence of *C. trachomatis* and HPV infection was 4.7% (236/5006) and 15.5% (778/5006), respectively. The prevalence of *C. trachomatis* infection was higher in the outpatients (5.2%) from GC than in those from PEC (3.8%) or ART (3.5%) (*P* = 0.043, Table 1), and was also higher in the group of ≤25 years than in those of >25 years (10.4 vs 4.0%, *P* < 0.001, Table 1 and Supplementary Table S1). The age-related difference of *C. trachomatis* infection was supported by multivariate analysis (Table 2) and existed among the participants from different departments (Supplementary Table S1). These results indicated that young women of less than 25 years and the patients in the gynecology clinic are the risk group for *C. trachomatis* infection.

**TABLE 1:** Prevalence of *C. trachomatis* and HPV infection of women in Southern Hunan province in China.

Characteristic	Sample size (*n*)	Age, *y* (Mean ± SD)	Prevalence [*n* (%)]		
			CT^+^	HPV^+^	CT^+^/HPV^+^
Total	5006	36.4 ± 10.7	236 (4.7)	778 (15.5)	59 (1.2)
**Clinical departments**					
PEC	1006	39.4 ± 11.1	38 (3.8)	109 (10.8)	6 (0.6)
ART	666	33.4 ± 5.5	23 (3.5)	69 (10.4)	5 (0.8)
Gynecology Outpatient	3334	36.1 ± 11.1	175 (5.2)	600 (18.0)	48 (1.4)
*P*			0.043	<0.001	0.053
**Age (years)**					
≤25	567	NA	59 (10.4)	121 (21.3)	22 (3.8)
>25	4439	NA	177 (4.0)	656 (14.8)	37 (0.8)
26–35	2122	NA	104 (4.9)	321 (15.1)	21 (1.0)
36–45	1222	NA	45 (3.7)	147 (12.0)	7 (0.6)
≥46	1095	NA	28 (2.6)	188 (17.2)	9 (0.8)
*P*			<0.001	<0.001	<0.001

*PEC, Physical Examination Center; ART, Assisted Reproductive Technology; CT, Chlamydia trachomatis; HPV, Human Papillomavirus; CT/HPV, Chlamydia trachomatis and Human Papillomavirus co-infection; NA, Not Applicable.*

**TABLE 2:** Factors associated with *Chlamydia trachomatis* or HPV infection by multivariate analysis.

Characteristic Ages, y	Odds Ratio (95% Confidence Interval)	P*	Characteristic Ages, y	Odds Ratio (95% Confidence Interval)	P*
≤25	1 (Reference)	<0.001	≤25	1 (Reference)	<0.001
26–35	0.496(0.354–0.697)		26–35	0.721(0.571-0.910)	
36–45	0.379(0.252–0.569)		36–45	0.557(0.427-0.726)	
≥46	0.240(0.151–0.383)		≥46	0.823(0.637-1.064)	

**HPV infection**			**CT infection**		

HPV^–^	1 (Reference)	<0.001	CT^–^	1 (Reference)	<0.001
HPV^+^	1.710 (1.254–2.332)		CT^+^	1.716(1.259-2.339)	

*By Hosmer-Lemeshow Satterthwaite adjusted F-test. CT, Chlamydia trachomatis; HPV, Human Papillomavirus.

Similar results were obtained for HPV infection, which was more often detected in the outpatients (18.0%) from GC than in those from PEC (10.8%) or ART (10.4%) (*P* < 0.001, Table 1), and in the group of ≤25 years than in those of >25 years (21.3 vs 14.8%, *P* < 0.001, Supplementary Table S1). However, the prevalence of HPV infection was not significantly different between the age group of >25 years and ≤25 years for those seeking annual physical examination (*P* = 0.156) and assistant reproduction treatment (*P* = 0.565, Supplementary Table S1).

### Co-infection of *C. trachomatis* and HPV

*C. trachomatis* and HPV coinfection was only detected in 1.2% (59/5006) of the participants and more often observed in women aged ≤25 years than in those of >25 years (3.8 vs 0.8%, *P* < 0.001). However, the prevalence of *C. trachomatis* and HPV co-infection was not significantly different among the women from PEC (0.6%), ARC (0.8%) and GC (1.4%) (*P* = 0.053) (Table 1).

Among the 236 participants infected with *C. trachomatis*, 59 (25.0%) were co-infected with HPV. We also noted a higher prevalence of *C. trachomatis* in HPV positive women (7.6%, 59/778) than HPV negative ones (4.2%, 177/4235) with an odds ratio of 1.72 (95% CI: 1.254–2.332, *P* < 0.001) (Table 2 and Supplementary Table S4). For the 778 participants infected with HPV, 59 (7.6%) were co-infected with *C. trachomatis*. The prevalence of HPV infection was 25.0% (59/236) in *C. trachomatis* positive women and 15.2% (719/4770) in *C. trachomatis* negative women with an odds ratio of 1.72 (95% CI: 1.259–2.339, *P* < 0.001) (Table 2 and Supplementary Table S5). These results indicate the tight association of *C. trachomatis* and HPV infection. Such an association was further confirmed by a case-control study in which *C. trachomatis* positive cases and controls were matched by age and population groups. We found that *C. trachomatis* infection was associated with high risk of HPV infection with an odds ratio of 1.74 (95% CI 1.10–2.74, *P* = 0.017). Similar results were observed for the enhanced *C. trachomatis* infection among HPV-infected women with an odds ratio of 1.69 (95% CI 1.10–2.59, *P* = 0.015) (Supplementary Table S4 and S6).

### Genotyping of *C. trachomatis* and HPV

The VS1–S2 fragment of *C. trachomatis* Omp1 gene was successfully amplified from 236 *C. trachomatis-positive* samples and 229 discernible sequences were classified into 8 genotypes. The top three *C. trachomatis* genotypes were E (1.4%, 69/5006), F (1.1%, 53/5006) and J (0.8%, 41/5006), accounting for 30.1, 23.1, and 17.9% of *C. trachomatis-positive* samples (Table 3). No significant difference was observed in the distribution of *C. trachomatis* genotypes among the participants from different clinical departments (*P* = 0.78, Table 3). Interestingly, one sample was originally classified as subtype B of *C. trachomatis* according to the Omp1 gene and was further confirmed by multilocus sequence typing (MLST) to be infected with subtype B (data not shown).

**TABLE 3:** Genotype distribution of 229 urogenital *Chlamydia trachomatis* stains by clinical departments.

CT genotypes	Clinical departments [*n* (%)]				*P*
	PEC	ART	Gynecology Outpatients	Total	
E	8 (21.6)	7 (31.8)	54 (31.8)	69 (30.1)	0.78
F	12 (32.4)	3 (13.6)	38 (22.4)	53 (23.1)	
J	6 (16.2)	5 (22.7)	30 (17.6)	41 (17.9)	
D	4 (10.8)	4 (18.2)	23 (13.5)	31 (13.5)	
G	3 (8.1)	1 (4.5)	10 (5.9)	14 (6.1)	
H	3 (8.1)	2 (9.1)	6 (3.5)	11 (4.8)	
K	1 (2.7)	0 (0)	8 (4.7)	9 (3.9)	
B	0 (0)	0 (0)	1 (0.6)	1 (0.4)	
Total	37 (100)	22 (100)	170 (100)	229 (100)	

*PEC, Physical Examination Center; ART: Assisted Reproductive Technology; CT: Chlamydia trachomatis.*

**FIGURE 1:**
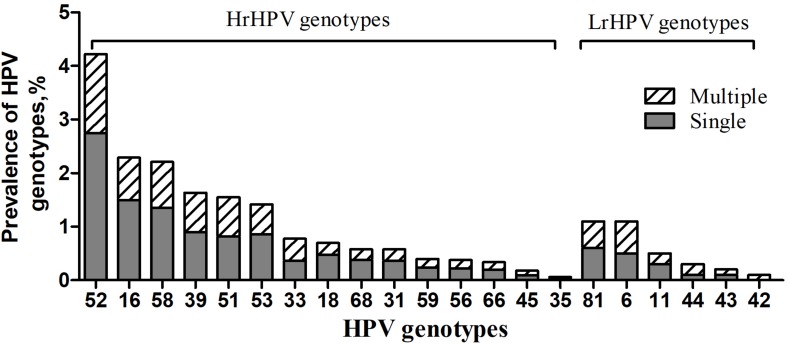
Prevalence of HrHPV and LrHPV genotypes in single and multiple HPV infections. The most common HrHPV genotype detected was HPV 52(4.2%), followed by HPV 16(2.3%), HPV 58(2.2%), HPV 39(1.6%), HPV 51(1.6%), HPV 53(1.4%), HPV 33(0.8%), HPV 18(0.7%), HPV 68(0.6%), HPV 31(0.6%), HPV 59(0.4%), HPV 56(0.4%), HPV 66(0.3%), HPV 45(0.2%) and HPV 35(0.1%). For the LrHPV genotypes, the most common type was HPV 83(1.1%), followed by HPV 6(1.0%), HPV 11(0.4%), HPV 44(0.3%), HPV 43(0.2%) and HPV 42(0.1%). HrHPV: High-risk HPV; LrHPV: Low-risk HPV.

Furthermore, the overall prevalence of different HPV genotype infection was as follows: 12.9% (648/5006) for HrHPV-only, 1.5% (77/5006) for LrHPV-only and 1.1% (53/5006) for the superinfection of HrHPV and LrHPV, respectively (Table 4). As shown in Figure 1, the most common HrHPV genotype was HPV52 (4.2%), followed by HPV16 (2.3%), HPV58 (2.2%), HPV39 (1.6%), HPV51 (1.6%), HPV53 (1.4%), HPV33 (0.8%), HPV18 (0.7%), HPV68(0.6%), HPV31 (0.6%), HPV59 (0.4%), HPV56 (0.4%), HPV66 (0.3%), HPV45 (0.2%) and HPV35 (0.1%). For the LrHPV genotypes, the most common type was HPV83 (1.1%), followed by HPV6 (1.0%), HPV11 (0.4%), HPV44 (0.3%), HPV43 (0.2%) and HPV42 (0.1%). There was no significant difference of both HrHPV and LrHPV genotypes distribution among different clinical departments (*P* = 0.45), various age groups (*P* = 0.61) and between *C. trachomatis* positive or negative women (*P* = 0.24, Table 4). However, HrHPV only infection was more often detected in *C. trachomatis* positive women (22.0%), outpatients in GC (14.6%) and young females of <25 years (16.2%) (Table 4), suggesting that these women may be more prone to HrHPV infection.

**TABLE 4:** Prevalence of low-risk and high-risk of HPV genotypes in 5023 women by *C. trachomatis* positivity, clinical departments and ages.

Characteristic	Sample Size (*n*)	HPV genotypes [*n* (%)]			*P*
		LrHPV only	HrHPV only	Mixed Hr/LrHPV	
Total	5006	77(1.5)	648(12.9)	53(1.1)	
***C. trachomatis* infection**					0.24
CT^–^	4770	71 (1.5)	596 (12.5	52 (1.1)	
CT^+^	236	6 (2.5)	52 (22.0)	1 (0.4)	
*P*		0.509	0.01	0.45	
**Clinical departments**					
PEC	1006	9 (0.9)	98 (9.7)	2 (0.2)	0.45
ART	666	7 (1.1)	60 (9.7)	2 (0.3)	
Gynecology Outpatient	3343	61 (1.8)	490 (14.6)	49 (1.5)	
*P*		0.018	<0.001	<0.001	
**Age, y**					
≤25	567	16 (2.8)	92 (16.2)	13(2.3)	0.61
26–35	2122	31 (1.5)	274 (12.9)	17 (0.8)	
36–45	1222	12 (1.0)	125 (10.2)	10 (0.8)	
≥46	1095	18 (1.6)	157 (14.3)	13 (1.2)	
*P*		<0.001	0.012	0.011	

*PEC: Physical Examination Center; ART, Assisted Reproductive Technology; CT, Chlamydia trachomatis; HPV, Human Papillomavirus; HrHPV, High-risk HPV; LrHPV, Low-risk HPV.*

Also, 77.4% (602/778) of HPV positive participants infected with only one HPV genotype while 17.4% (136/778) and 4.6% (36/778) of them infected with 2 and 3 HPV genotypes, respectively. There were only 5 cases (0.6%) infected with more than 4 HPV genotypes (Supplementary Table S2). We noted a significant difference in the clinical department-specific distribution of single and duplicate genotype infections (*P* < 0.01 and *P* < 0.05). HPV52 (137, 17.6%) accounted for the most common HrHPV genotypes in those with a single genotype of HPV infection, followed by HPV16 (74, 9.5%), and HPV58 (68, 8.6%) (Supplementary Table S3). The difference of HPV52 prevalence was also documented in women from PEC, ART, and GC, which is 23.9, 27.5, and 15.2%, respectively.

### Factors Associated With *C. trachomatis* and HPV Infection

The variables associated with *C. trachomatis* infection statistically significant were age (*P* < 0.01) and HPV infection (*P* < 0.01, Table 1 and Supplementary Table S4), which were further confirmed by the multivariate analysis (Table 2). However, *C. trachomatis* infection was not associated with single or multiple HPV infections (*P* > 0.05), or the genotypes of HPV (*P* > 0.05, Supplementary Table S4).

As shown in Table 2, HPV infection was independently associated with age (*P* < 0.01) and *C. trachomatis* infection (*P* < 0.01, Table 2). However, there was no correlation between *C. trachomatis* and HPV genotype distribution among participants with successful genotyping of both pathogens (*P* > 0.05) (Supplementary Table S7).

### Associations of Colposcopy Impression Features With *C. trachomatis* and HPV Infection

To investigate the association between cervical abnormalities and *C. trachomatis*/HPV infection, colposcopy data were collected from 151 outpatients from GC. Cervical abnormal features were found in 37.7% (57/151) of the patients while low and high grade features, as well as cervical cancer, accounted for 75.4% (43/57), 17.5% (10/57), and 7.0% (4/57), respectively (Table 5). For HPV-infected outpatients, the prevalence of low and high grade features as well as cervical cancer was 42.6% (29/68), 11.8% (8/68), and 5.9% (4/68), respectively (Table 5). Of note, out of 4 cancer patients further confirmed by pathological reports (data not shown), 3 were infected with HrHPV and 1 with LrHPV. Only low and high grade features were observed in 38.2% (13/34) and 5.9% (2/34) of *C. trachomatis*-infected patients, respectively (Table 5).

**TABLE 5:** Cytology-associated *C. trachomatis* and HPV infection among 151 Cases.

	Sample Size (*n*)	Colposcopy impression [*n* (%)]			*P*
		Benign	Low grade	High grade	
CT and HPV infection	151	94	43	14	
CT^+^/HPV^+^	9	3 (33.3)	6 (66.7)	0	<0.001
CT^–^/HPV^–^	58	51 (87.9)	7 (12.1)	0	
CT^+^/HPV^–^	25	16 (64.0)	7 (28.0)	2 (8.0)	
CT^–^/HPV^+^	59	24 (40.7)	23 (39.0)	12 (20.3)	
*P*		<0.001	<0.001	<0.001	
HPV infection	151	94	43	14	
LrHPV	8	3 (37.5)	4 (50.0)	1 (12.5)	<0.001
HrHPV	53	23 (43.4)	23 (43.4)	7 (13.2)	
Mixed HrHPV and LrHPV	7	1 (14.3)	2 (28.6)	4 (57.1)	
Negative	83	67 (80.7)	14 (16.9)	2 (2.4)	
*P*		<0.001	0.044	<0.001	

*CT, Chlamydia trachomatis; HPV, Human Papillomavirus.*

Furthermore, HPV infection was strongly associated with an abnormal colposcopy impression of both low-grade (OR: 6.872, 95% CI: 2.894–16.317, *P* < 0.001) and high-grade (OR: 15.863, 95% CI: 3.144–80.03, *P* < 0.001, Supplementary Table S8). However, our study showed that CT infection was only associated with low-grade colposcopy abnormality with OR of 3.248 (95% CI: 1.22–8.646, *P* = 0.018).

## Discussion

The prevalence and genotype distribution of *C. trachomatis* and HPV in women differ considerably between nations and regions even within a country. Li and colleagues have reported that the prevalence of *C. trachomatis* and HPV, as well as *C. trachomatis*/HPV coinfection was 14.3, 36.0, and 4.8%, respectively in Northern part of Inner Mongolia, China (Ji et al., 2019). Nearly 2.2% of married women were found to be infected with *C. trachomatis*, and 7.0% of them were infected with high risk HPV in Beijing, China (Zhang et al., 2017). In our study, we found that the prevalence of *C. trachomatis*, HPV, and *C. trachomatis*/HPV coinfection was 4.7, 15.5, and 1.2%, respectively in the Southern part of Hunan, China. To our best knowledge, this is the first large observational investigation into *C. trachomatis* and HPV infection in women of Southern China. Our results support the prevalence of *C. trachomatis* and HPV among various areas in China and highlight the importance of epidemiological studies in different regions for the big area and huge population size.

Both *C. trachomatis* and HPV are characterized by symptomless infection. We found that the prevalence of asymptomatic *C. trachomatis*, HPV, and *C. trachomatis*/HPV co-infection was 3.8% (38/1006), 10.8% (109/1006), and 0.6% (6/1006), respectively in women attending annual physical examination who are believed to be healthy general females, although a slightly higher prevalence of *C. trachomatis* and HPV infection was documented in outpatients from GC. These findings highlighted the importance of asymptomatic infection of *C. trachomatis* and HPV infection in the general population, particularly in young women of less than 25 years. These results are consistent with previous findings and further support that young women should be the target population for the prevention of *C. trachomatis* and HPV (Samoff et al., 2005; Bellaminutti et al., 2014). Importantly, women from ART had a relatively high prevalence of *C. trachomatis* (3.5%) and HPV (10.4%) infection, especially among women with secondary infertility (Shi et al., 2001), indicating the necessary *C. trachomatis* and HPV screening for women of childbearing age.

In our study, only 1.2% of the participants were co-infected with *C. trachomatis* and HPV. Furthermore, 25.0% of *C. trachomatis* infection and 7.6% of HPV infection were attributable to *C. trachomatis* and HPV coinfection. Multivariate analysis also indicated that *C. trachomatis* or HPV infection increased the risk of HPV or *C. trachomatis* co-infection. These results were further confirmed by a case-control study in which the potential confounding factors of age and sources of the participants were matched. Besides, we also found both *C. trachomatis* and HPV infection were associated with colposcopy impression. These results suggest that C. trachomatis and HPV may act as mutual risk factors for their infection, and increase the risk of cervical lesions (Naldini et al., 2019). An alternative explanation for the tight association of *C. trachomatis* and HPV infection could also be their shared infection routes (Ji et al., 2019).

Interestingly, we found that the overall HPV-positive rate was 15.5%, which was relatively lower than our previous estimated prevalence of 30.16% in 2009 and that of 16.8–36% reported in other studies in China (Li et al., 2006; Ji et al., 2019). The decreasing prevalence of HPV infection was probably due to expanding sexual safety education, and continuous improvements of living conditions and health in the past decade in China, although the sensitivities of testing assays may be different among the studies. Notably, 14% of the participants were positive for HrHPV while only 2.6% were positive for LrHPV, indicating the seriousness of high-risk genotypes of HPV infection and cervical cancer. Of note, our results were lower than the average HrHPV infection rate of 19% reported by Li and colleagues (Li et al., 2019).

Several studies have described HPV-16 as the most prevalent genotype in China (Bellaminutti et al., 2014). However, the genotype distribution of HPV in Southern Chinese women appears to be different (Ji et al., 2019). We found that HPV52 (4.2%), HPV16 (2.3%) and HPV58 (2.2%) were the three dominant HrHPV genotypes in women. These results are in line with the data from other Chinese populations in which both HPV52 and 58 are the predominant type among the general population in China (Bi et al., 2015; Wang et al., 2015). In Eastern Asia, infection of HP52 and 58 also resulted in 2.5–2.8- and 3.7–4.9-fold higher risk for cervical intraepithelial neoplasia (CIN) and invasive cancer than in other regions (Chan et al., 2014). The HPV vaccine is an important strategy for the prevention of cervical cancer and has been implemented for more than 10 years. However, HPV vaccination was launched until July 2017 in mainland China. Moreover, the first domestic bivalent HPV vaccine was licensed by the China Food and Drug Administration (CFDA) on Jan, 2020. Currently, very few Chinese women have been vaccinated due to a variety of reasons including inaccessible vaccine, economic, educational, and so on. Our results indicate that future HPV vaccine should cover HPV genotype 52, 58, and 16 when used in Southern part of China.

We found that serotype E (30.1%), F (23.1%) and J (17.9%) were the top three *C. trachomatis* serovars in the current study. Similar results were found in other parts of China (Yang et al., 2010), with serotype E being predominant in the South (32%) and East (27%), and F in the Southwest (28%) in China (Hsu et al., 2006; Gao et al., 2007). While in Thailand, serotype F (35%) and E (18%) was the most prevalent genotypes whereas F (32%) and E (17%) dominated in Japan, and F (31%) and E (23%) in Netherlands (Yamazaki et al., 2005).

We noted no significant associations between *C. trachomatis* and HPV genotype distribution by using correlation analysis, and also found no particular associations of Lr- and HrHPV genotypes, and single and multiple HPV infections with *C. trachomatis* infection in the general population, indicating that type-specific deviations from the overall association between *C. trachomatis* and HPV acquisition are small. Nevertheless, an association between *C. trachomatis* and HrHPV genotypes in female adolescents has been demonstrated previously (Samoff et al., 2005). Again, this discrepancy could be possibly explained by the geographic and within-population variation, and the small sample size of women with coinfections in the current study.

It is worth mentioning that *C. trachomatis* serovars E, D, and J account for 70%, and HPV 52, 51 and 39 for 36% of coinfection cases. This distribution of HPV genotypes contrasts with the report that HPV16 was the most represented in women without *C. trachomatis* coinfection (Seraceni et al., 2016), but in agreement with the recent Italian data that described these genotypes as the most frequent types associated with coinfections (Carozzi et al., 2014) in which HPV 52, 51, and 39 of groups 1, 2 A or 2 B have been classified as cervical carcinogens (Clarke et al., 2018). More interestingly, HPV 52 was also the genotype that is most frequently detected among women from ART, which reinforced the notion that HPV 52 was most commonly found in couples undergoing in vitro fertilization treatment, and in the case of low sperm concentration and reduced sperm motility, as recently presented from a Lithuanian report (Jersoviene et al., 2019).

Some limits, however, existed in this study: (1) the participants were from a hospital, not from a community population. Thus, the results might not reflect the infection of *C. trachomatis* and HPV in the general population; (2) lack of detailed information on the history of women with *C. trachomatis* or HPV infection and histological changes prevented the identification of the risk factors for *C. trachomatis* and HPV infection; and (3) nucleic acid detection of *C. trachomatis* and HPV by PCR only reflects current infection. The prevalence of *C. trachomatis* and HPV may be underestimated (Moscicki et al., 1998).

In conclusion, a large observational study was conducted to investigate the prevalence and genotype distribution of both *C. trachomatis* and HPV in women in the Southern part of China. Overall, the prevalence of *C. trachomatis* and HPV infection was similar in healthy women attending physical examination or seeking reproductive assistance to the outpatients GC, indicating the importance of asymptomatic *C. trachomatis* and HPV infection, particularly among females aged less than 25 years. *C. trachomatis* or HPV infection could increase the risk of HPV or *C. trachomatis* co-infection. These observations highlight the need for routine *C. trachomatis* and HPV screening and monitoring.

## Data Availability Statement

The raw data supporting the conclusions of this article will be made available by the authors, without undue reservation, to any qualified researcher.

## Ethics Statement

The studies involving human participants were reviewed and approved by the Ethics Committee of Chenzhou No. 1 People’s Hospital. The patients/participants provided their written informed consent to participate in this study.

## Author Contributions

ZL, ST, and HC designed the study. HC, LL, YW, BH, and HL contributed to CT and HPV detection. XH and PH did the colposcopy test. ZL, XH, PH, and LL helped with data management. HC and JS contributed to statistical analysis. HL and ST drafted the manuscript. All authors commented on the draft and decision to submit.

## Conflict of Interest

The authors declare that the research was conducted in the absence of any commercial or financial relationships that could be construed as a potential conflict of interest.

## Footnotes

1 http://www.ncbi.nlm.nih.gov/BLAST
