# Supplementary material for: Chlamydia trachomatis and Human Papillomavirus Infection in Women From Southern Hunan Province in China: A Large Observational Study
Source: Front Microbiol. 2020 May 5;11:827. doi: 10.3389/fmicb.2020.00827 (PMC7214719; doi:10.3389/fmicb.2020.00827)
Supplement: Supplementary file 1 [file Data_Sheet_1.docx]

**Supplementary data:**

**Table S1. Age-stratified prevalence of *Chlamydia trachomatis*, HPV and *Chlamydia trachomatis*/HPV Coinfection**

| **Age, y** | **PEC [n (%)]** | | |  | **ART [n (%)]** | | |  | **Gynecology Outpatient [n (%)]** | | |  | **Total [n (%)]** | | |
| --- | --- | --- | --- | --- | --- | --- | --- | --- | --- | --- | --- | --- | --- | --- | --- |
|  | **Sample**  **Size(n)** | **CT^+^** | **HPV^+^** |  | **Sample**  **Size(n)** | **CT^+^** | **HPV^+^** |  | **Sample**  **Size(n)** | **CT^+^** | **HPV^+^** |  | **Sample**  **Size(n)** | **CT^+^** | **HPV^+^** |
| ≤25 | 64 | 8 (10.2) | 11 (17.2) |  | 35 | 3 (8.6) | 2 (5.7) |  | 468 | 48 (10.3) | 108 (23.1) |  | 567 | 59 (10.4) | 121 (21.3) |
| >25 | 942 | 30 (3.2) | 98 (10.4) |  | 631 | 20 (3.2) | 67 (10.6) |  | 2866 | 127 (4.4) | 491 (17.1) |  | 4439 | 177 (4.0) | 656(14.8) |
| *P* |  | 0.003 | 0.156 |  |  | 0.127 | 0.565 |  |  | <0.001 | 0.005 |  |  | <0.001 | <0.001 |

PEC: Physical Examination Center; ART: Assisted Reproductive Technology; CT: *Chlamydia trachomatis*; HPV: Human Papillomavirus.

**Table S2. Distribution of single and multiple HPV infections in 778 urogenital HPV stains by clinical departments and ages**

| **Characteristic** | **HPV Infection [n (%)]** | | | | |
| --- | --- | --- | --- | --- | --- |
|  | **Single HPV**  **genotype** | **Double HPV**  **genotypes** | **Triple HPV**  **genotypes** | **Quadruple HPV**  **genotypes** | **Quintuplicate**  **HPV genotypes** |
| **Total** | 602(77.4) | 135(17.4) | 36(4.6) | 4(0.5) | 1(0.1) |
| **Clinical departments** |  |  |  |  |  |
| PEC | 92(84.4) | 12(11) | 4(3.7) | 1(0.9) | 0(0) |
| ART | 61(88.4) | 7(10.1) | 1(1.4) | 0(0) | 0(0) |
| Gynecology Outpatient | 449(74.8) | 116(19.3) | 31(5.2) | 3(0.5) | 1(0.2) |
| *P* | 0.006 | 0.03 | 0.31 | 0.69 | 0.86 |
| **Age, y** |  |  |  |  |  |
| ≤25 | 83(68.6) | 28(23.1) | 8(6.6) | 2(1.7) | 0(0) |
| 26-35 | 262(81.2) | 51(15.7) | 8(2.8) | 1(0.3) | 0(0) |
| 36-45 | 114(77.6) | 26(17.7) | 7(4.8) | 0(0) | 0(0) |
| ≥46 | 143(76.1) | 30(16) | 13(6.9) | 1(0.5) | 1(0.5) |
| *P* | 0.058 | 0.33 | 0.14 | 0.28 | 0.36 |

PEC: Physical Examination Center; ART: Assisted Reproductive Technology; HPV: Human Papillomavirus.

**Table S3. Genotype distribution of 778 urogenital HPV stains by clinical departments**

| **Characteristic** | | **Clinical departments [n (%)]** | | | | ***P*** |
| --- | --- | --- | --- | --- | --- | --- |
|  |  | **PEC** | **ART** | **Gynecology Outpatients** | **Total** |  |
| **HPV genotypes** | |  |  |  |  |  |
| **HrHPV** | |  |  |  |  |  |
| 52 | | 26(23.9) | 19(27.5) | 92(15.3) | 137(17.6) | 0.75 |
| 16 | | 8(7.3) | 8(11.6) | 58(9.7) | 74(9.5) |  |
| 58 | | 10(9.2) | 10(14.5) | 48(7.9) | 68(8.6) |  |
| 39 | | 11(10.1) | 1(1.4) | 33(5.4) | 45(5.7) |  |
| 53 | | 6(5.5) | 5(7.2) | 32(5.2) | 43(5.5) |  |
| 51 | | 7(6.4) | 3(4.3) | 31(5.1) | 41(5.2) |  |
| 18 | | 3(2.8) | 1(1.4) | 20(3.3) | 24(3) |  |
| 68 | | 2(1.8) | 2(2.9) | 15(2.5) | 19(2.4) |  |
| 33 | | 2(1.8) | 0(0) | 16(2.6) | 18(2.3) |  |
| 31 | | 1(0.9) | 2(2.9) | 15(2.5) | 18(2.3) |  |
| 59 | | 1(0.9) | 2(2.9) | 9(1.5) | 12(1.5) |  |
| 56 | | 2(1.8) | 1(1.4) | 8(1.3) | 11(1.4) |  |
| 66 | | 2(1.8) | 0(0) | 7(1.3) | 9(1.3) |  |
| 45 | | 2(1.8) | 0(0) | 3(0.5) | 5(0.6) |  |
| 35 | | 0(0) | 0(0) | 1(0.2) | 1(0.1) |  |
| **2+ HrHPV only** | | 15(13.8) | 6(8.7) | 99(16.2) | 120(15.2) | 0.88 |
| **Lr/urHPV** | |  |  |  |  |  |
| 81 | | 2(1.8) | 4(5.8) | 23(3.8) | 29(3.7) | 0.32 |
| 6 | | 1(0.9) | 1(1.4) | 20(3.3) | 22(2.8) |  |
| 11 | | 2(1.8) | 0(0) | 10(1.6) | 12(1.5) |  |
| 43 | | 2(1.8) | 0(0) | 2(0.3) | 4(0.5) |  |
| 44 | | 2(1.8) | 1(1.4) | 2(0.3) | 5(0.6) |  |
| 42 | | 0(0) | 1(1.4) | 1(0.2) | 2(0.3) |  |
| **2+ LrHPV only** | | 0(0) | 0(0) | 3(0.5) | 3(0.4) | 0.98 |
| **Mixed HrHPV and Lr/urHPV** | 2(1.8) | | 2(2.9) | 49(8.2) | 53(6.8) | 0.018 |

PEC: Physical Examination Center; ART: Assisted Reproductive Technology; HPV: Human Papillomavirus; HrHPV: High-risk HPV; LrHPV: Low-risk HPV.

**Table S4. Prevalence of *Chlamydia trachomatis* infection by ways of HPV infection.**

| **Characteristic** | **CT infection[n (%)]** | | ***P*** |
| --- | --- | --- | --- |
|  | **CT^+^** | **CT^-^** |  |
| **HPV infection** |  |  |  |
| HPV^+^ | 59 (7.6) | 719(92.4) | <0.001 |
| HPV^-^ | 177 (4.2) | 4051(95.8) |  |
| **HPV infection status** |  |  |  |
| Single infection | 46(7.8) | 556(92.2) | 0.91 |
| Multiple infection | 13(7.4) | 163(92.6) |  |
| **HPV genotypes*** |  |  |  |
| LrHPV | 6(7.8) | 71(92.2) | 0.24 |
| HrHPV | 52(8.0) | 596(92.0) |  |
| Mixed HrHPV and LrHPV | 1(1.9) | 52(98.1) |  |
| **HPV 52*** |  |  |  |
| HPV 52^+^ | 10(7.2) | 128(92.8) | 0.98 |
| HPV 52^-^ | 49(7.5) | 600(92.5) |  |
| **HPV 16*** |  |  |  |
| HPV 16^+^ | 4(5.3) | 71(94.7) | 0.76 |
| HPV 16^-^ | 55(7.7) | 657(92.3) |  |

*778 urogenital HPV positive women. CT: *Chlamydia trachomatis*; HPV: Human Papillomavirus; HrHPV: High-risk HPV; LrHPV: Low-risk HPV.

**Table S5. Prevalence of HPV infection by ways of *Chlamydia trachomatis* infection.**

| **Characteristic** | **HPV infection[n (%)]** | | ***P*** |
| --- | --- | --- | --- |
|  | **HPV^+^** | **HPV^-^** |  |
| **CT infection** |  |  |  |
| CT^+^ | 59(25.0) | 177(75.0) | <0.001 |
| CT^-^ | 719(15.2) | 4051(84.8) |  |
| **CT E genotype*** |  |  |  |
| CT E^+^ | 6(7.1) | 79(92.9) | 0.24 |
| CT E^-^ | 52(8.0) | 596(92.0) |  |
| **CT F genotype*** |  |  |  |
| CT F^+^ | 10(7.2) | 128(92.8) | 0.98 |
| CT F^-^ | 49(7.5) | 601(92.5) |  |

*229 urogenital *Chlamydia trachomatis* positive women. CT: *Chlamydia trachomatis*; HPV: Human Papillomavirus.

**Table S6. Associations between *Chlamydia trachomatis* and HPV infection in a case-control study matched by age and clinical department**

| **Parameters** | **CT infection[n (%)]** | | ***P*** | **Parameters** | **HPV infection[n (%)]** | | ***P*** |
| --- | --- | --- | --- | --- | --- | --- | --- |
|  | **CT^+^(cases)** | **CT^-^ (controls)** |  |  | **HPV^+^ ( cases)** | **HPV^-^ ( controls)** |  |
| **Age, y (Mean ± SD)** | 32.42 ± 9.38 | 32.62 ± 9.90 | 0.823 | **Age, y (Mean ± SD)** | 36.01 ± 11.60 | 36.22 ± 11.24 | 0.714 |
| **Clinical departments** | n=236 | n=236 | 1 | **Clinical departments** | n=788 | n=788 | 1 |
| PEC | 38 (16.1) | 38 (16.1) |  | PEC | 109 (13.8) | 109 (13.8) |  |
| ART | 23 (9.7) | 23 (9.7) |  | ART | 69 (8.8) | 69 (8.8) |  |
| Gynecology Outpatient | 175 (74.2) | 175 (74.2) |  | Gynecology Outpatient | 610 (77.4) | 610 (77.4) |  |
| **HPV DNA^*^** | n=236 | n=236 | 0.017 | **CT DNA^#^** | n=788 | n=788 | 0.015 |
| Positive | 59 (25) | 38 (16.1) |  | Positive | 59 (7.5) | 36 (4.6) |  |
| Negative | 177(75) | 198 (83.9) |  | Negative | 729 (92.5) | 752 (95.4) |  |

By Chi-Square Tests (Pearson Chi-Square, Continuity Correction), *OR=1.74 (1.10-2.74) and ^#^OR=1.69 (1.10-2.59). CT: *Chlamydia trachomatis*; HPV: Human Papillomavirus.

**Table S7.** Genotype distribution of *C. trachomatis* and HPV among women with *Chlamydia trachomatis*/HPV Coinfection

| **HPV genotypes** | **CT genotypes(n)** | | | | | | | | ***P**** | **Spearman’s coefficient** | |
| --- | --- | --- | --- | --- | --- | --- | --- | --- | --- | --- | --- |
|  | **E** | **D** | **J** | **F** | **G** | **H** | **K** | **Total** |  | **R** | ***P*** |
| 52 | 1 | 5 | 2 | 2 | 0 | 0 | 0 | 10 | 0.67 | 0.08 | 0.61 |
| 51 | 2 | 2 | 0 | 1 | 1 | 0 | 0 | 6 |  |  |  |
| 39 | 0 | 2 | 1 | 1 | 0 | 1 | 0 | 5 |  |  |  |
| 58 | 1 | 0 | 2 | 0 | 0 | 1 | 0 | 4 |  |  |  |
| 16 | 1 | 0 | 1 | 1 | 1 | 0 | 0 | 4 |  |  |  |
| 6 | 2 | 1 | 1 | 0 | 0 | 0 | 0 | 4 |  |  |  |
| 53 | 0 | 0 | 2 | 1 | 0 | 0 | 0 | 3 |  |  |  |
| 18 | 2 | 0 | 0 | 1 | 0 | 0 | 0 | 3 |  |  |  |
| 68 | 0 | 0 | 0 | 0 | 1 | 0 | 0 | 1 |  |  |  |
| 59 | 1 | 0 | 0 | 0 | 0 | 0 | 0 | 1 |  |  |  |
| 56 | 0 | 1 | 0 | 0 | 0 | 0 | 0 | 1 |  |  |  |
| 33 | 1 | 0 | 0 | 0 | 0 | 0 | 0 | 1 |  |  |  |
| 31 | 1 | 0 | 0 | 0 | 0 | 0 | 0 | 1 |  |  |  |
| 81 | 0 | 0 | 0 | 1 | 0 | 0 | 0 | 1 |  |  |  |
| Multiple infection | 4 | 1 | 3 | 3 | 0 | 0 | 2 | 13 |  |  |  |

*By Wald χ2 statistic. CT: *Chlamydia trachomatis*; HPV: Human Papillomavirus.

**Table S8. *C. trachomatis* and HPV infections as risk factors for colposcopic impression among women in southern China**

|  | **CT infection OR^2^**  **(95% CI)** | ***P**** | **HPV infection OR^1^**  **(95% CI)** | ***P**** |
| --- | --- | --- | --- | --- |
| **Benign** | 1 (Reference) |  | 1 (Reference) |  |
| **Low grade** | 3.248(1.22-8.646) | 0.018 | 6.872(2.894-16.317) | <0.001 |
| **High grade** | 1.663(0.291-9.492) | 0.567 | 15.863(3.144-80.03) | <0.001 |

*****By Hosmer-Lemeshow Satterthwaite adjusted F test. OR^1^ = odds ratio—adjusted for age and HPV infection; OR^2^—adjusted for age and *C. trachomati*s infection; CT: *Chlamydia trachomatis*; HPV: Human Papillomavirus.
